# The Different Physiological and Antioxidative Responses of Zucchini and Cucumber to Sewage Sludge Application

**DOI:** 10.1371/journal.pone.0157782

**Published:** 2016-06-21

**Authors:** Anna Wyrwicka, Magdalena Urbaniak

**Affiliations:** 1 University of Lodz, Faculty of Biology and Environmental Protection, Department of Plant Physiology and Biochemistry, Lodz, Poland; 2 University of Lodz, Faculty of Biology and Environmental Protection, Department of Applied Ecology, Lodz, Poland; 3 European Regional Centre for Ecohydrology of the Polish Academy of Sciences, Lodz, Poland; Hainan University, CHINA

## Abstract

The present study investigates the effect of soil amended with sewage sludge on oxidative changes in zucchini and cucumber plants (*Cucurbitaceae*) and the consequent activation of their antioxidative systems and detoxification mechanisms. The plants were grown in pots containing soil amended with three concentrations of sewage sludge (1.8 g, 5.4 g and 10.8 g per pot), while controls were potted with vegetable soil. The activities of three antioxidative enzymes, ascorbate peroxidase (APx), catalase (CAT) and guaiacol peroxidase (POx), were assessed, as well as of the detoxifying enzyme S-glutathione transferase (GST). Lipid peroxidation was evaluated by measuring the extent of oxidative damage; α-tocopherol content, the main lipophilic antioxidant, was also measured. Visible symptoms of leaf blade damage after sewage sludge application occurred only on the zucchini plants. The zucchini and cucumber plants showed a range of enzymatic antioxidant responses to sewage sludge application. While APx and POx activities increased significantly with increasing sludge concentration in the zucchini plants, they decreased in the cucumber plants. Moreover, although the activity of these enzymes increased gradually with increasing doses of sewage sludge, these levels fell at the highest dose. An inverse relationship between peroxidases activity and CAT activity was observed in both investigated plant species. In contrast, although GST activity increased progressively with sludge concentration in both the zucchini and cucumber leaves, the increase in GST activity was greater in the zucchini plants, being visible at the lowest dose used. The results indicate that signs of sewage sludge toxicity were greater in zucchini than cucumber, and its defense reactions were mainly associated with increases in APx, POx and GST activity.

## Introduction

The production of sewage sludge is steadily increasing, and with it the importance of the question of its safe and sustainable disposal, as it often contains a variety of toxic compounds. These pollutants include inorganic compounds such as heavy metals, and organic chemicals, such as Polycyclic Aromatic Hydrocarbons (PAHs) and other Persistent Organic Pollutants (POPs) characterized by high persistence, potential for bioaccumulation, biomagnification, and toxicity [1–6]. Consequently, the storage and utilization of such deposits present a risk for the environment, and specific methods are required to ensure safe disposal.

One such method is phytoremediation, which is based on the use of physiological processes of vegetation and microorganisms to extract, degrade, stabilize and/or remove contaminants from given matrices [7–9]. However, the plants used in phytoremediation not only need a high capacity to absorb pollutants, but also should be resistant to the stress caused by their presence. Plant-based techniques are inexpensive and socially acceptable, can be applied on a large scale and in most cases are carried out successfully. Although phytoremediation techniques are known to be capable of removing heavy metals [10–12], the process of organic compound phytoremediation still requires elucidation. The key problems are associated with the hydrophobicity of organic compounds (log K_ow_ between 5.0 to 8.3). Studies have shown that substances with a log K_ow_ greater than 3.5 are not available to plants, since they are strongly adsorbed by soil particles and do not pass into the soil solution from which they could be taken up [13–14]. However, an exception is the group of plants belonging to the *Cucurbitaceae*, including among others, the cucumber, squash and pumpkin [15–17]. This group of plants has the potential to accumulate higher levels of organic compounds in their tissues [5, 17–30], and the accumulated compounds have been found to originate from the soil rather than the air [15].

The use of sludge contaminated with a range of inorganic and organic compounds as fertilizer may affect the development and physiology of the plants grown on it. On the one hand, the addition of sewage sludge to soil creates better conditions for plant growth by improving the physicochemical properties of soils, such as total organic carbon content, cation exchange capacity and microbiological profile [31–32]. On the other hand, repeated application of sewage sludge increases not only the heavy metal content in soil, but also its uptake and accumulation in plant tissues [33]. Therefore, a number of studies have assessed the methods used to reduce sludge toxicity, such as composting the sludge with plant material [32], the use of surfactant amendment [6] or conversion of sludge to sewage sludge biochar [34]. Most current studies regarding the impact of sewage sludge on crops concern mainly biomass growth or improvement of soil conditions. There are few papers addressing the biochemical changes caused by the application of sewage sludge.

Many worldwide studies report that abiotic stresses, such as the presence in soil of inorganic compounds, such as heavy metals [35], or organic compounds, such as PCBs and PCDD/F [5], or the presence of osmotic stress [36], UV radiation [37] and acid rain [38–40] are the main causes of secondary oxidative stress [41]. This phenomenon, occurring in plant tissues, is characterized with overproduction of Reactive Oxygen Species (ROS) such as superoxide anions (O_2_^-·^), hydroxyl radicals (·OH) and peroxyl radicals (ROO·), singlet oxygen (^1^O_2_) and hydrogen peroxide (H_2_O_2_). These molecules are highly reactive and may cause the oxidation of important cellular compounds, resulting in lipid peroxidation and irreversible protein degradation, as well as reduced plant growth and development, by damaging nucleic acids and other biologically important molecules. Although ROS are produced as a natural phenomenon in plant tissues which accompanies photosynthesis and respiration [42], the balance between their production and disposal is disturbed under oxidative stress.

One symptom of lipid damage is the presence of elevated levels of the end products of polyunsaturated fatty acid oxidation. These compounds react with thiobarbituric acid (*thiobarbituric acid-reactive-substances*; TBARS) to form a chromogen. TBARS concentration is reliable marker of lipid peroxidation. Plants possess extensive and efficient enzymatic and non-enzymatic antioxidant defense systems which operate to control the cascade of uncontrolled oxidation and to protect plant cells from oxidative damage by scavenging ROS. Antioxidative enzymes such as superoxide dismutase (SOD; EC 1.15.1.1) [43] or catalase (CAT; EC 1.11.1.6) [44] can directly remove ROS, while such others as ascorbate peroxidase (APx; EC 1.11.1.1) or glutathione reductase (GR; 1.8.1.7), related to the Halliwell-Asada cycle, work using low molecular weight antioxidants [45]. Another group of antioxidant enzymes comprises phenolic peroxidases, e.g. guaiacol peroxidase (POx; EC 1.11.1.7) [46]. These enzymes are present in the plant cell wall in soluble forms. The peroxidases involved in the defense response are responsible for the stiffening of the cell wall. The non-enzymatic system includes antioxidants such as ascorbic acid [47], glutathione and other non-protein thiol groups [48], phenolic compounds [49], carotenoids and α-tocopherol [50].

Glutathione S-transferase (GST; EC 2.5.1.18) [51] is one of the biotransformation enzymes which take part in the second phase of xenobiotic detoxification and catalyse conjugation of the chemical pollutant to an endogenous substrate: reduced glutathione. Plant GSTs were discovered as a part of herbicide detoxification in terrestrial plants but many authors have demonstrated their involvement in the detoxification of polycyclic aromatic hydrocarbons (PAHs), PCBs and heavy metals [5, 12, 52].

This study evaluates the physiological response of two plant species belonging to the *Cucurbitaceae* family, zucchini and cucumber, to the compounds present in sewage sludge. To identify differences between the two investigated species, the following characteristics were examined: morphological change, degree of oxidative reaction assessed as TBARS content, and the concentration of α-tocopherol, the main lipophilic, non-enzymatic antioxidant. In addition, the study measures the enzymatic activities of APx, CAT and POx to determine the efficiency of the antioxidative system, and uses GST activity to evaluate the performance of detoxification reactions.

## Materials and Methods

### Soil preparation

Sewage sludge from the Lodz Municipal Wastewater Treatment Plant (LM WWTP) was collected. It was dried at 70°C for 72 hours, then homogenized into small particles with a mortar and used as soil fertilizer for zucchini and cucumber cultivation. The information regarding the content of toxic substances in control soil and in the soil amended with sewage sludge at dose of 9 t·ha^-1^ is presented in Table 1. The vegetable potting soil (specific for the above vegetable growth) used in the experiment was from Hollas Sp. z o.o. Pasłęk. Sewage sludge was obtained from the resources of the Lodz Wastewater Treatment Plant (location: Sanitariuszek 66, 93–469 Łódź, Central Poland) as part of a research and scientific cooperation between the Wastewater Treatment Plant, the University of Lodz and the European Regional Centre for Ecohydrology of the Polish Academy of Sciences, Lodz. Our experiment was carried out in the growth chambers in the laboratory of the Department of Plant Physiology and Biochemistry, University of Lodz.

**Table 1:** Physico-chemical properties of the untreated and sewage sludge amended soil at the dose of 9 t·ha^-1^.

Properties	Unit	Control	Soil mixed with 9 t/ha of sewage sludge
pH		6.02+/-0.4	6.4+/-0.4
Total Organic Carbon	g/kg	15.18	22.67
Total Nitrogen	%	0.52+/-0.08	0.66+/-0.10
N-NO_3_	mg/kg d.w.	782+/-234	859+/-257
P	mg/kg d.w.	437+/-88	728.7+/-146
K	mg/kg d.w.	2341+/-445	2809+/-534
Ca	mg/kg d.w.	2640+/-501	2755+/-523
Mg	mg/kg d.w.	1099	1221
Cl	mg/kg d.w.	69.4+/-17.4	127.3+/-31.8
Na	mg/kg d.w.	150	190
Pb	mg/kg d.w.	5.7+/-1.4	7.1+/-1.8
Cd	mg/kg d.w.	<0.21	<0.21
Cr	mg/kg d.w.	<4.2	31.3+/-6.4
Cu	mg/kg d.w.	22.2+/-4.6	31.3+/-6.4
Ni	mg/kg d.w.	4.9+/-1.1	6.4+/-1.5
Zn	mg/kg d.w.	22.4+/-4.2	72.8+/-13.6
Fe	mg/kg d.w.	2297+/-430	3311+/-619
Mn	mg/kg d.w.	115+/-23.7	112.3+/-23.1

Four treatments were used: a control (C) in which no sludge was added, and three treatments of 1.8 g, 5.4 g and 10.8 g sewage per pot. The first corresponds to a dose of 3 tonnes ha^-1^ yr^-1^ permitted by the Regulation of the Minister of the Environment dated 6 February 2015 concerning municipal sewage sludge (Dz.U. Nr 2015 r., poz. 257); the second is equivalent to 9 tonnes, the permitted amount for three years applied in one dose; and the third, 18 tonnes ha^-1^ yr^-1^ is above the permitted level. Treatments are designated by the numerical dose per pot.

### Plant material

Zucchini (*Cucurbita pepo* L.) cv “Atena Polka” and cucumber seeds (*Cucumis sativus* L.) cv “Cezar” were germinated in Petri dishes for seven days and the seedlings were planted into either control or sewage sludge-amended soil. They were grown in a growth chamber at 23 ± 0.5°C with 16 h light/8 h dark cycle with 250 μmol m^-2^ s^-1^ photon flux density during the light period and 60% relative humidity. Three-week old zucchini plants and five-week old cucumber plants with five fully expanded leaves were used for subsequent analysis. All biochemical analyses were carried out on the second, third and fourth leaves from the control and treated plants. The leaves were harvested in the middle of the 16 h light period.

### Preparation of enzyme extracts from leaf tissues

The leaves of the zucchini and cucumber plants were ground (1:10, w/v) in an ice-cold mortar using 50 mM sodium phosphate buffer (pH 7.0) containing 0.5 M NaCl, 1 mM EDTA, and 1 mM sodium ascorbate. The slurry was filtered through two layers of Micracloth. The filtrates of homogenized zucchini and cucumber leaves were then centrifuged (15000g x 15 min). After centrifugation, the supernatant was collected and APx, CAT, GST and POx activities as well as protein concentration and degree of lipid peroxidation were measured.

### Enzyme assay

APx activity [EC 1.11.1.11] was assayed following the oxidation of ascorbate to dehydroascorbate at 265 nm (ε = 13.7 mM^**−**1^ cm^**−**1^) according to Nakano and Asada with some modifications [53]. The assay mixture contained 50 mM sodium phosphate buffer pH = 7.0, 0.25 mM sodium ascorbate, 25 μM H_2_O_2_ and the enzyme extract (5–10 μg protein). The addition of H_2_O_2_ started the reaction. The obtained values were compared with those of another reaction mixture without the enzyme extract to correct for non-enzymatic oxidation of ascorbate. The enzyme activity was expressed in nkat mg^-1^ protein.

CAT activity [EC 1.11.1.6] was measured spectrophotometrically according to Dhinsa et al. [43]. A reaction mixture composed of 50mM sodium phosphate buffer (pH = 7.0), 15 mM H_2_O_2_ and the enzyme extract (5–10 μg protein) was used. The decomposition of H_2_O_2_ (ε = 45.2 mM^**−**1^ cm^**−**1^) was measured at 240 nm. CAT activity was expressed in μkat mg^-1^ protein.

The total GST activity [EC 2.5.1.18] was determined with 1-chloro-2,4-dinitrobenzene (CDNB) according to Habig et al. with some modification [54]. GST catalyses the conjugation of L-glutathione (GSH) to CDNB through a thiol group of GSH. The product of CDNB conjugation with GSH, dinitrophenyl thioether, absorbs at 340 nm (ε = 9.6 mM^−1^ cm^−1^). The reaction solution contained 100 mM potassium phosphate buffer (pH 6.25), 0.75 mM CDNB, 30 mM GSH and the enzyme extract (50 μg protein). The enzyme activity was expressed in nkat mg^-1^ protein.

POx activity [EC 1.11.1.7] was assayed with guaiacol according to Maehly and Chance, with modifications, [55]. The reaction mixture contained 49 mM sodium acetate buffer (pH 5.6) 5 mM guaiacol, 15 mM H_2_O_2_, and the enzyme extract (15–25 μg protein). A linear increase in absorbance at 470 nm was observed due to the formation of tetraguaiacol (the millimolar extinction coefficient of tetraguaiacol at 470 nm; ε = 26.6 mM^−1^ cm^−1^). This was monitored for four minutes at 25°C. The enzyme activity was expressed in μkat mg^-1^ protein.

All assays were performed spectrophotometrically (UNICAM UV 300 UV-visible spectrometer) at 25°C.

### Degree of lipid peroxidation (TBARS)

Concentration of lipid peroxides was estimated spectrofluorometrically according to Yagi [56] by measuring the content of 2-thiobarbituric acid reactive substances (TBARS). The concentration of lipid peroxides was calculated in terms of 1,1,3,3-tetraethoxypropane, which was used as a standard and expressed in nmol g^-1^ fresh mass.

### Protein content

The protein content was determined by Bradford’s method [57] with standard curves prepared using bovine serum albumin.

### Determination of α-tocopherol

Whole leaves were homogenized (1:5 w/v) in an ice-cold mortar using 50 mM sodium phosphate buffer, pH 7.0, containing 0.5 M NaCl, 1 mM EDTA and 1 mM sodium ascorbate. Crude homogenate obtained after filtration was assayed for α-tocopherol content according to Taylor et al. [58]. After saponification of the sample with KOH in the presence of ascorbic acid, α-tocopherol was extracted with n-hexane. Fluorescence of the organic layer was measured at 280 nm (excitation) and 310 nm (emission) using a F-2500 Fluorescence Spectrophotometer (Hitachi, Limited, Tokyo Japan). The concentration of α-tocopherol was expressed as μg g^-1^ fresh mass of the original plant tissue.

### Statistical analysis

All measurements were performed in two replicates in three to four independent experiments (*n* = 6–8). Variation is given as the standard deviation (S.D.) of means. The nonparametric Mann-Whitney Rank Sum Test was used to evaluate the significance of differences between mean values. *P* = 0.05 was taken as the level of significance.

## Results

**Fig 1:**
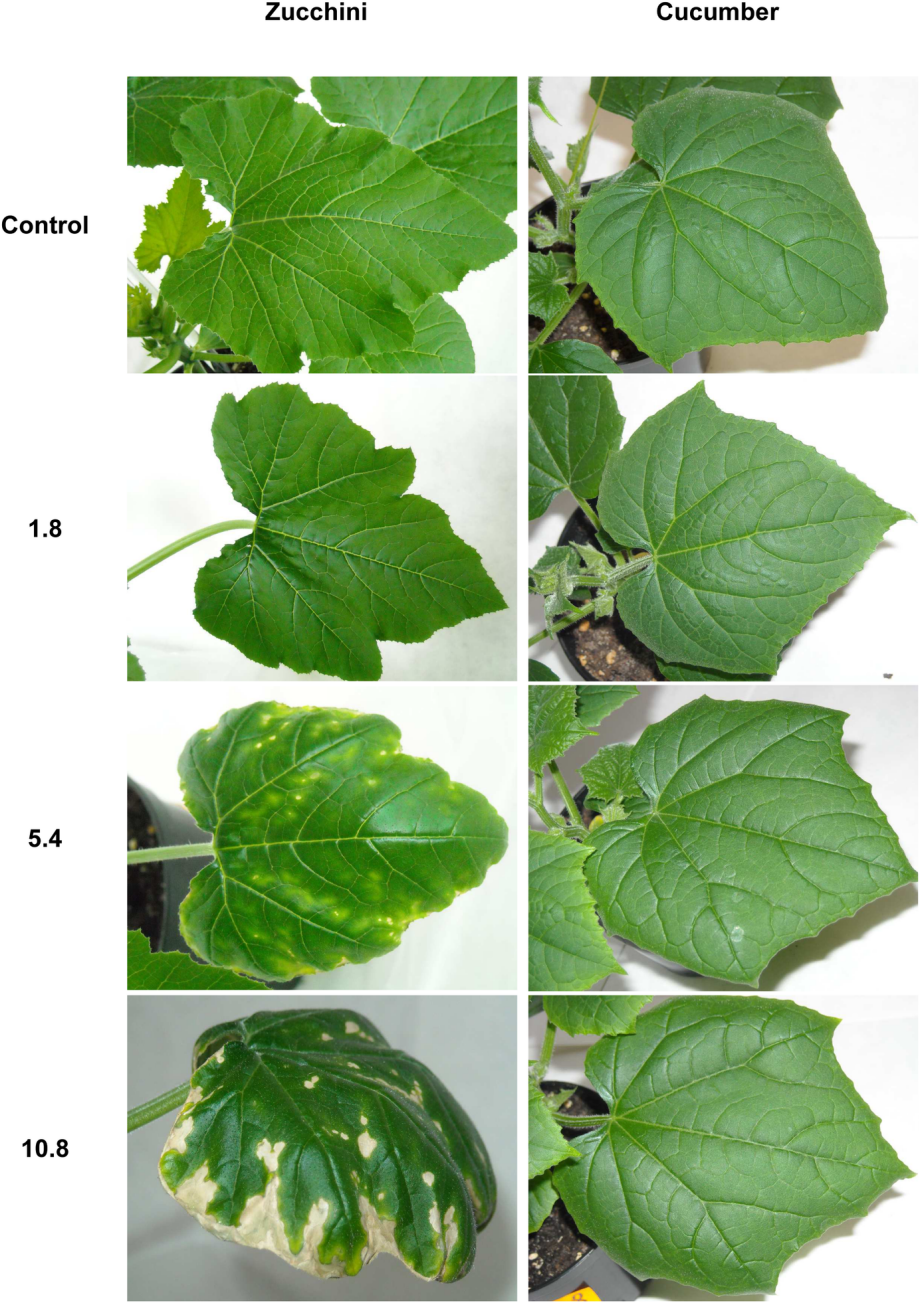
Visible injuries on leaves of zucchini and cucumber grown on the control and sewage sludge amended soil.

Some visible symptoms in the form of small protrusions of leaf surface were observed on zucchini plants immediately after the application of the smallest dose of sewage sludge (Fig 1). Although this dose did not cause any other morphological changes, the two higher doses produced more severe, visible damage.

Chlorotic spots and bleaching appeared in some parts of the leaf surface, particularly within the ribs, for the plants treated with the medium dose (5.4g). These changes were found most commonly on the oldest leaves and the intensity of changes increased with leaf age. Additionally, the edges of the leaves demonstrating these changes were slightly rolled downward and the surface was distorted between the leaf ribs.

At the highest dose (10.8g), the changes were similar but more intense: the white spots on the leaves were more numerous, necrotic changes appeared particularly on the edges of the leaves and the leaves themselves became umbrella-shaped. Moreover, the plants were much smaller than controls. In contrast to zucchini, no signs of damage were seen on cucumber plants regardless of the variant.

**Fig 2:**
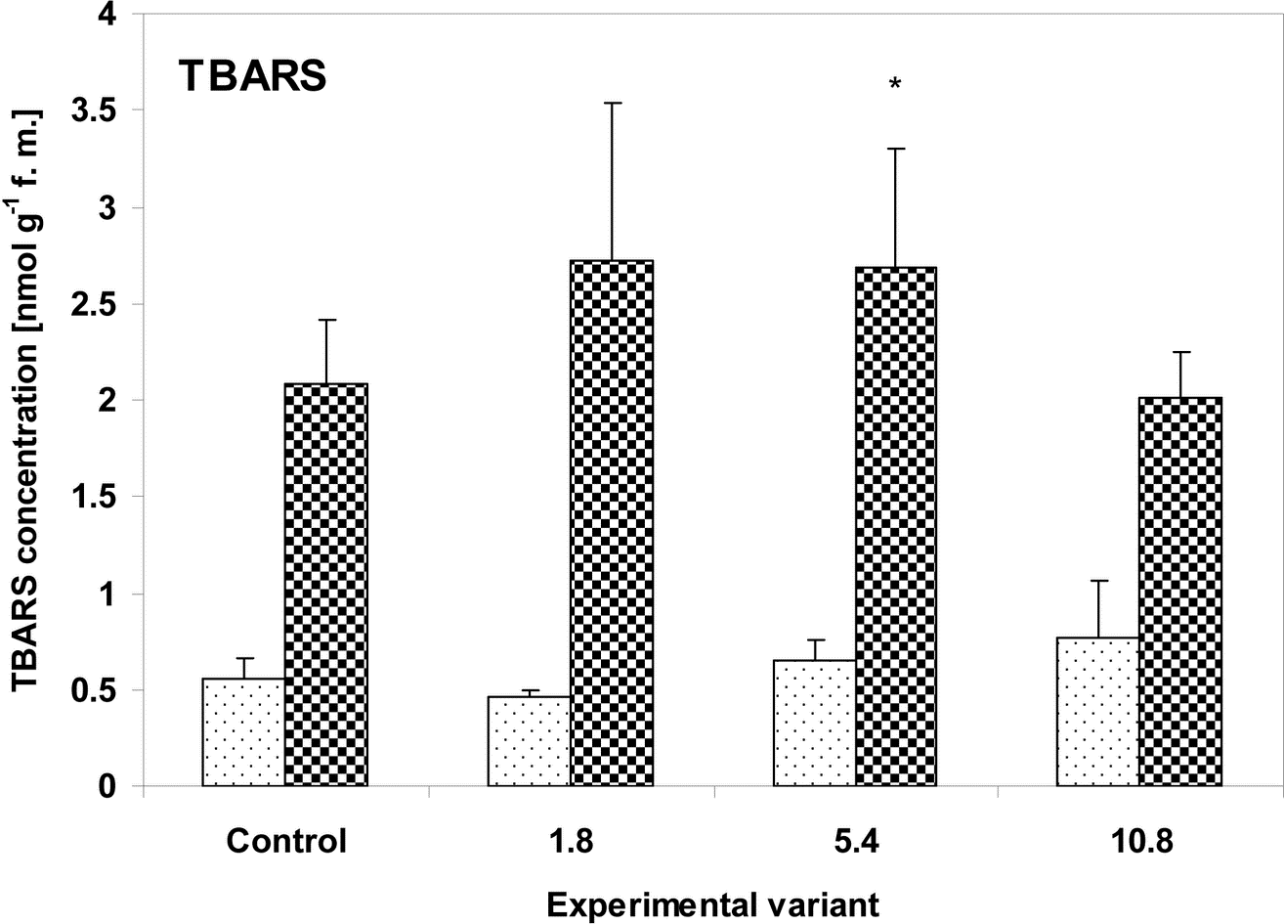
TBARS concentration in the leaves of zucchini (square without filling) and cucumber (square with dots) grown on the control and sewage sludge amended soil. Concentrations are expressed in nmol g^-1^ f.m. Bars represent S.D. of means (*n* = 5–8). Symbols (*), (**), and (***) indicate values that differ significantly from controls at *P*<0.05, *P*<0.01, and *P*<0.001, respectively. Activities are expressed in nkat mg^-1^ protein.

Sewage sludge application did not cause significant changes in TBARS concentration, an indicator of oxidative lipid damage, in the zucchini nor the cucumber plants (Fig 2). TBARS concentrations were slightly higher at medium and high doses (5.4g and 10.8g) in the zucchini leaves and in the low and medium doses (1.8g and 5.4g) in cucumber. However, the changes were statistically significant only in the cucumber leaves at the medium dose of 5.4g (129% of the control value, P<0.05).

**Fig 3:**
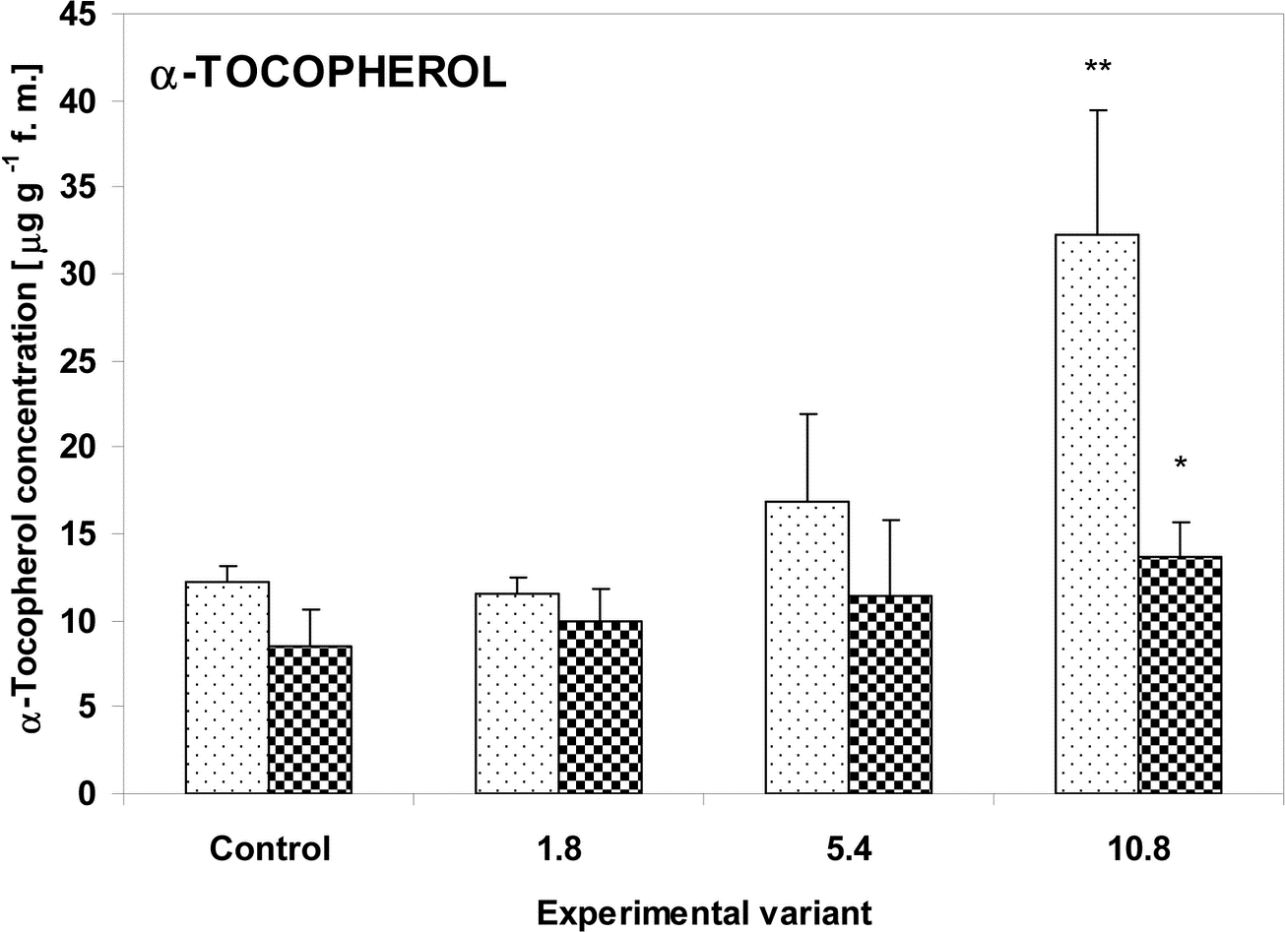
α-Tocopherol concentration in the leaves of zucchini (square without filling) and cucumber (square with dots) grown on the control and sewage sludge amended soil. Concentrations are expressed in μg g^-1^ f.m. For further explanation, see Fig 2.

The concentration of α-tocopherol, a lipophilic antioxidant, increased with sewage sludge addition both in the zucchini and cucumber leaves. However, significant changes were observed only for the maximum dose of 10.8g: 265% (P<0.01) for zucchini and 160% (P<0.05) for cucumber, compared with the control (Fig 3).

**Fig 4:**
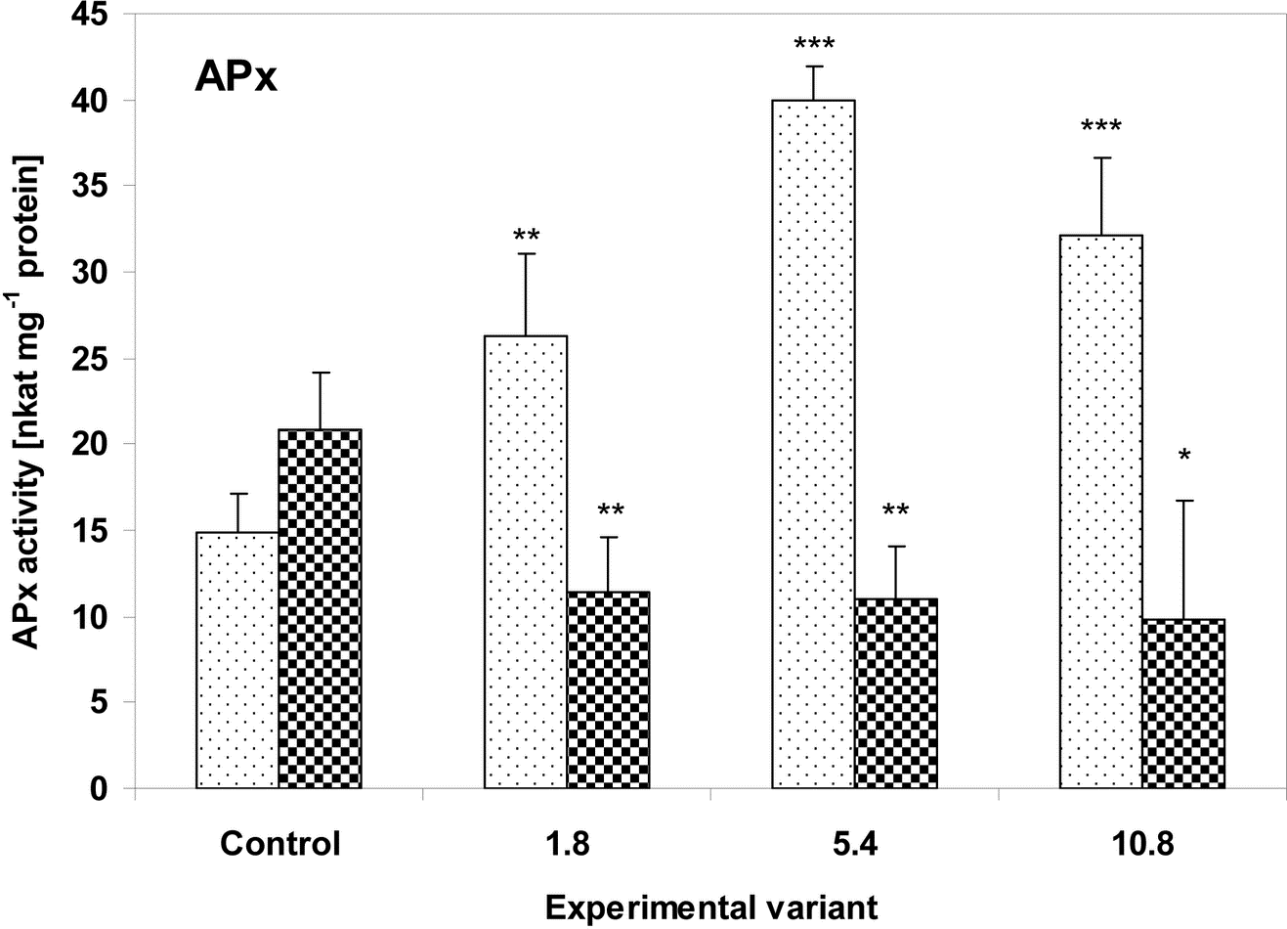
Activity of APx in the leaves of zucchini (square without filling) and cucumber (square with dots) grown on the control and sewage sludge amended soil. For further explanation, see Fig 2.

The nature of the changes observed for the antioxidative enzyme system was closely connected with both the investigated plant species and with the dose of sewage sludge. In the leaves of the zucchini plants, APx activity was highest at the medium dose (5.4g) (270% of the control value; P<0.001); however it was still significantly higher than controls at the other doses: 178% for 1.8g (P<0.01) and 217% for 10.8g (P<0.001) (Fig 4). In contrast, the cucumber plants demonstrated significant decreases to 55% (P<0.01), 53% (P<0.01) and 47% (P<0.05) for doses of 1.8g, 5.4g and 10.8g, respectively.

**Fig 5:**
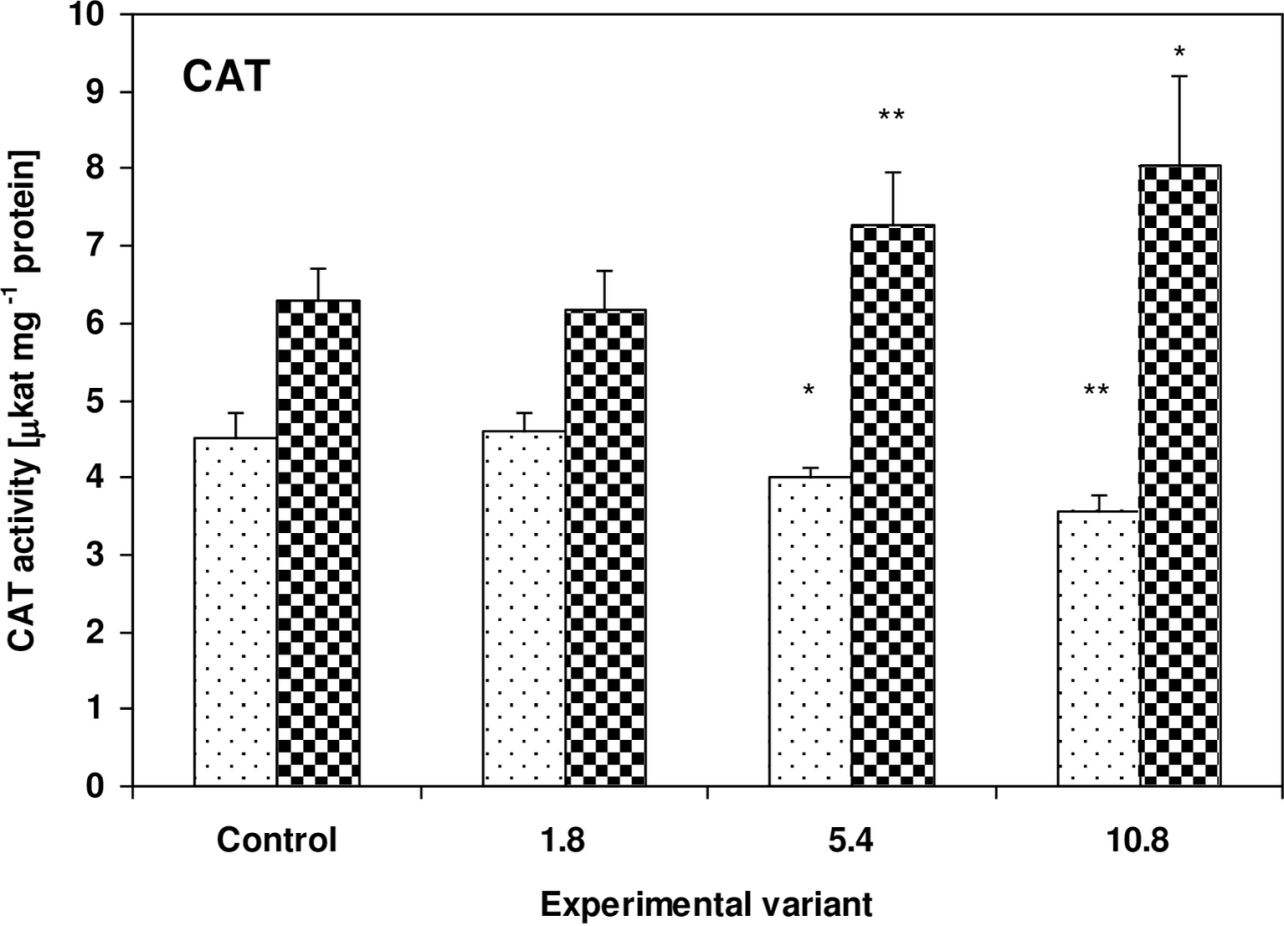
Activity of CAT in the leaves of zucchini (square without filling) and cucumber (square with dots) grown on the control and sewage sludge amended soil. Activities are expressed in μkat mg^-1^ protein. For further explanation, see Fig 2.

CAT activity showed the opposite tendency. While it decreased to 89% (P<0.05) and 79% (P<0.01) of control values in the zucchini leaves, it increased to 116% (P<0.01) and 128% (P<0.05) of control values in the cucumber leaves for the 5.4g and 10.8g doses, respectively (Fig 5).

**Fig 6:**
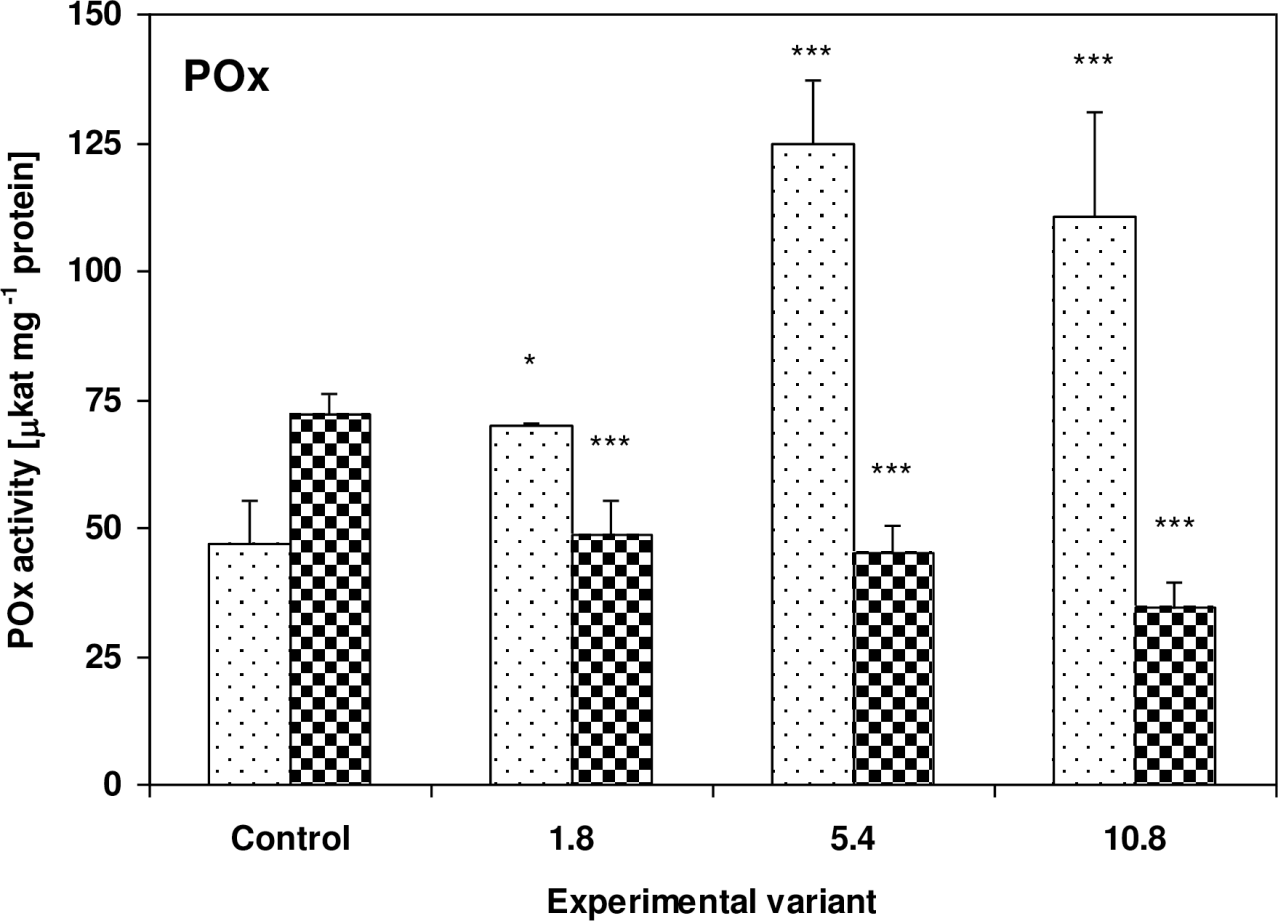
Activity of POx in the leaves of zucchini (square without filling) and cucumber (square with dots) grown on the control and sewage sludge amended soil. Activities are expressed in μkat mg^-1^ protein. For further explanation, see Fig 2.

POx activity increased in the zucchini leaves to 149% (P<0.05), 265% (P<0.001) and 234% (P<0.001) of control values, and decreased to 67% (P<0.001), 63% (P<0.001) and 48% (P<0.001) of controls in the cucumber leaves, for the 1.8g, 5.4g and 10.8g doses, respectively (Fig 6).

**Fig 7:**
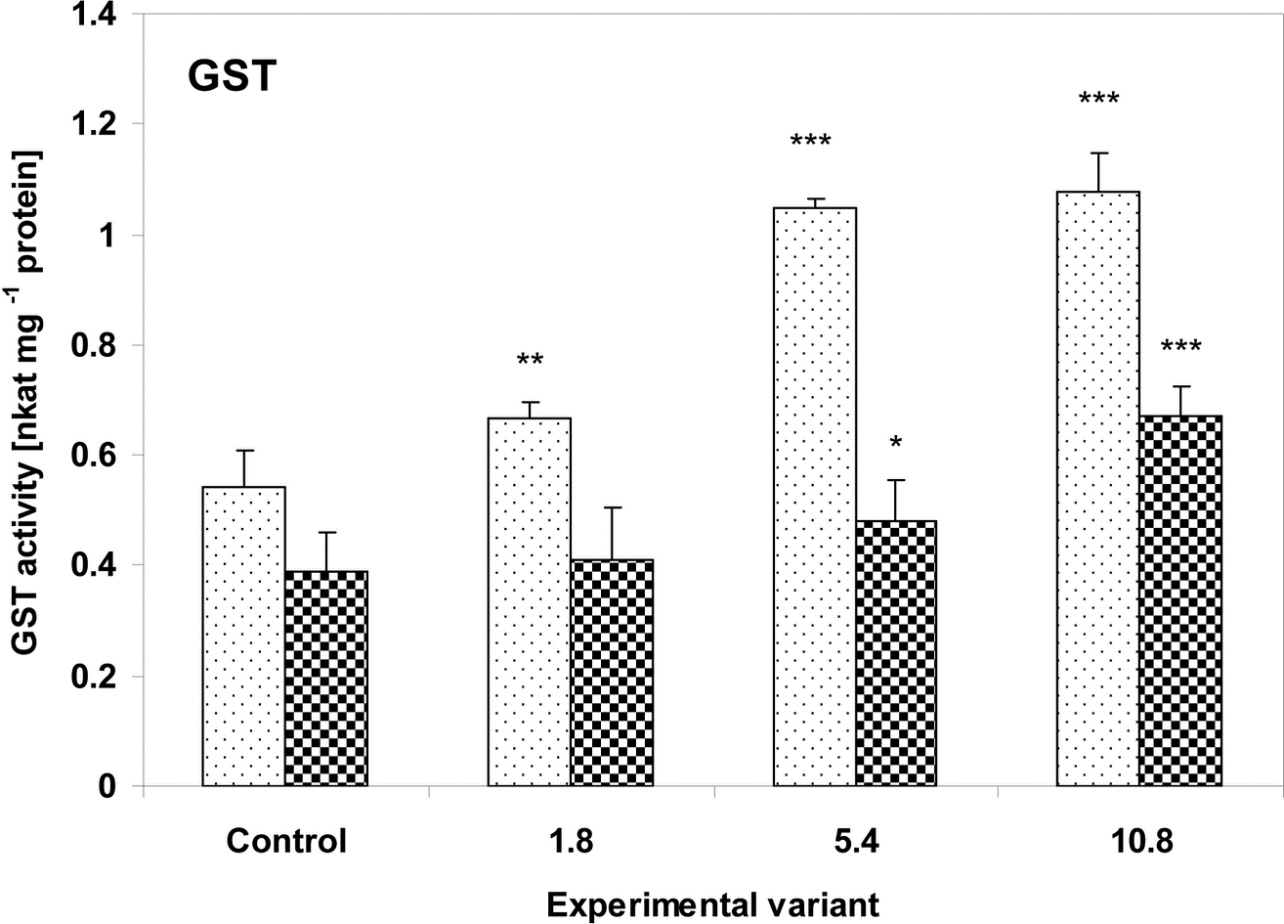
The activity of GST in the leaves of zucchini (square without filling) and cucumber (square with dots) grown on the control and sewage sludge amended soil. Activities are expressed in nkat mg^-1^ protein. For further explanation, see Fig 2.

The activity of GST, one of the main detoxifying enzymes, was increased in the leaves of both investigated plants. In the zucchini plants, GST activity increased even after the addition of the smallest dose of sludge, reaching 123% (P<0.01), 193% (P<0.001) 199% (P<0.001) of control values for the 1.8g, 5.4g and 10.8g doses respectively. In the cucumber leaves, GST activity was found to be 124% (P<0.05) and 175% (P<0.01) of control values for the 5.4g and 10.8g doses, respectively (Fig 7).

## Discussion

Although abiotic stresses are known to have a significant influence on plants, the impact of sewage sludge supplementation on plant growth and oxidative damages remains poorly understood, as does antioxidative response. Our findings demonstrate the different responses to sewage sludge application by zucchini and cucumber, two members of the *Cucurbitaceae* family. Due to its high organic matter and nutrient content, sewage sludge is regarded as an excellent fertilizer; however, the presence of associated micropollutants can hamper this positive influence on plant growth and development.

Our findings indicate that sewage sludge has a stronger effect on the zucchini than the cucumber plants, as demonstrated by the changes observed on the leaves after sewage sludge application. This difference could be attributed to either an increased sensitivity of zucchini plants to the substances present in the sewage sludge, or to a greater uptake of toxic compounds from the soil. In fact, some studies report that zucchini plants take up persistent organic pollutants (POPs) such as PCDD, PCDF and PCB from soil to a greater extent than cucumber plants [15, 23–24]. Many reports have identified discrepancies between members of the *Curbitaceae* with regard to their uptake of hydrophobic components: for example, zucchini were found to be far more contaminated with chlordecone than cucumber plants grown in the same medium [59]. Moreover, there is evidence that zucchini can accumulate metals such as zinc and cadmium to a similar extent as spinach, which is known to be an effective bioaccumulator of heavy metals [60]. Hence, the morphological changes such as white spots or protrusions observed on the zucchini leaf blades in the present study (Fig 1) may well be the result of higher accumulation of toxic substances from the applied sewage sludge than demonstrated by the cucumber plants. For effective phytoremediation of pollutants present in sewage sludge, it is crucial to use plants that have the ability to take up, translocate and accumulate the toxic substances it contains.

Environmental stresses may cause excessive ROS formation, which has the potential to interact with many cellular components, resulting in *inter alia* damage to membrane lipids and proteins. Many reports indicate that oxidative stress is an important component of the plant response to various toxic chemicals such as heavy metals [35], insecticides [61] or herbicides [62]. The concentration of TBA-reactive substances is one indicator of the degree of lipid oxidative damage. Although damage to the zucchini leaves was visible, only a slight increase in TBARS concentration was found in the tissues (Fig 2). This greater degree of lipid peroxidation appeared only in the 5.4g and 10.8g sewage samples, and these doses correlated with the severity of changes observed on the leaves. However, the concentration of α-tocopherol, the main antioxidant of the lipid fraction, also increased significantly in these variants, particularly for the maximum (10.8g) dose (Fig 3); this reflects its protective role, which is associated with preventing the formation of lipid peroxides from cellular lipids. In contrast to zucchini, the cucumber plants did not show any morphological changes nor any progressive increase in TBARS related to stress severity; however, the variant administered the highest dose of sewage sludge (10.8g) did display elevated levels of α-tocopherol. It is possible that in the case of cucumber plants, the antioxidant defense was not sufficient to completely block the formation of lipid peroxides at the 5.4g dose. For the 10.8g sample, the increase in α-tocopherol concentration was much lower in cucumber (163% of control) than zucchini plants (267% of control).

The type of technology used for sludge treatment is another factor with a significant impact on the oxidative damage experienced by the plants and their antioxidative response. The application of anaerobic mesophillic digested (AM) and autothermal thermophilic aerobic digested (ATAD) sludge on alfalfa (*Medicago sativa* L.) plants caused a significant increase in the concentration of malondialdehyde (MDA), a major cytotoxic product of lipid peroxidation, in the underground plant organs but not in the leaves [63]. Supplementation with AM sludge, the type used in the present study, tends to result in a lesser degree of oxidative stress in plant tissues than ATAD sludge. Moreover, the ability to scavenge ROS excess appears to be more successful in plants treated with AM than those with ATAD sludge. It is possible that the small range of lipid oxidative damage observed in the tested zucchini and cucumber plants may be due to the fact that AM technology was used to treat the sludge. It has been found that refinery sludge inhibited the growth of alfalfa and induced oxidative stress, but had little impact on lipid peroxidation [64], while another study showed that in alfalfa plants, the application of sewage sludge was associated with a significant reduction in the degree of oxidative damage to lipids during drought, as well as H_2_O_2_ concentration [65]. It is possible that the wide range of nutrients present in the sludge exert a beneficial influence by reducing the effect of toxic substances that can cause oxidative stress, and consequently, oxidative damage.

On the other hand, the degree of lipid peroxidation (TBARS content) in *Sinapis alba*, *Triticum aestivum*, *Phaseolus vulgaris* and *Vicia faba* seedlings was found to clearly increase in the presence of pollutants such as 1,2,4 –trichlorobenzene, carbazole, fluorene or phenanthridine. In these cases, TBARS content could be considered a biomarker for this kind of soil contamination [66–67] and serve as an early warning of phytotoxicity *in vivo*.

One defensive strategy used by plants to counter the effects of oxidative stress is based around adjusting the response to the environmental stressors through changes in the activity of antioxidant enzymes. The zucchini and cucumber plants investigated in this study showed a diversity of enzymatic antioxidant responses to sewage sludge application. While APx activity in the zucchini plants increased significantly after the application of sewage sludge, it decreased in the cucumber plants (Fig 4). Moreover, similar changes were observed in the tested plants with regard to POx activity (Fig 6). The activities of APx and POx in the leaves of zucchini plants changed in a concentration-dependent manner: rising with increasing quantities of sewage sludge, reaching their highest value in the 5.4g sample, and then falling in the highest sewage sludge dose (10.8g). Nevertheless, the values observed at 10.8g were still higher than controls.

A similar trend in APx activity, first increasing and then declining, was also observed in *Arabidopsis thaliana* plants treated with increasing phenantrene concentrations [68]. Both APx and glutathione peroxidase activity changed in the same way in *Arabidopsis thaliana* plants exposed to 2,3,7,8-tetrachlorinated dibenzo-*p*-dioxin [69]. A similar situation concerning APx and POx was also found in phenol-treated *Arabidopsis thaliana* plants [70]. It is possible that the decline in APx and POx activities observed at the maximum sewage sludge dose (10.8g) indicates that the production of toxic ROS may have overwhelmed the plant antioxidant systems at this dose, causing the observed damage to tissue structures.

In both the zucchini and cucumber plants, an inverse relationship was observed between the activity of peroxidases and the activity of CAT. While in the zucchini plants, APx and POx activity rose and CAT activity fell with increasing amounts of sewage sludge, the opposite situation was observed in the cucumber plants (Figs 4–6). It is worth noting that the morphological changes appeared only on the zucchini leaves. It cannot be excluded that, in the investigated plants, this behavior may be associated with a model of antioxidant response where APx and GST activities rise and CAT decreases. It is possible that despite the transient increase in TBARS concentration, morphological changes on the leaves of cucumber plants were prevented by increased CAT activity, and stressor-triggered biochemical changes tend to appear much earlier in plant tissues than visible toxicity symptoms. On the other hand, CAT activity is highly sensitive to the presence of many toxic substances; for example cyanide, azide, hydroxylamine, 3-amino-1,2,4-triazole, mercaptoethanol, urea and H_2_O_2_ have been found to act as CAT inhibitors [71–73]. As zucchini plants have been found to be capable of more efficient absorption of substances from the soil, including toxic substances, it seems possible that the presence of absorbed substances or their metabolites in tissues may inhibit CAT activity. In addition, CAT is not capable of decomposing H_2_O_2_ if the compound is present at concentrations too low for APx activity, due to its high Michaelis constant (K_m_) [71]. H_2_O_2_ acts as a signaling molecule: it initiates a signal transduction leading to enzyme activation or the expression of genes encoding such proteins as GST and POx [74]. A rapid decline of CAT mRNA observed the phenanthrene-treated *Arabidopsis thaliana* plants has been attributed to an attempt to increase H_2_O_2_ signaling and induce PCD [68]. Similar changes have also been observed after O_3_ and Cd ^2+^ stress [75–76]. Previous studies suggest that reduction of CAT activity is a distinctive, non-specific reaction of plants to stress factors such as low temperature [77], salinity [78], water deficit [79], SO_2_ fumigation [80], herbicides [81] and acid rain [82].

Similar trends were seen in GST activity in both zucchini and cucumber leaves; it increased gradually with the quantity of sewage sludge used. However, the increase in GST activity was higher in the zucchini plants and was observed even at the lowest administered dose. Increased GST activity in plant tissues indicates exposure to xenobiotics [83]. GST catalyzes the conjugation of electrophilic substrates with reduced glutathione, resulting in the formation of products which are more polar and less toxic [52, 84]. In other studies, GST activity has been found to significantly increase after exposure to a range of pollutants, including PAHs [67], hexachlorobenzene [83] and atrazine [85], and during detoxification of heavy metals [86–88]. The activity of GST was fond to be significantly elevated in the aquatic macrophyte *Ceratophyllum demersum* when exposed to low 3-chlorobiphenyl concentrations [89]. However, reduced GST activity was observed at higher 3-chlorobiphenyl levels, suggesting that its capacity may be limited.

Among the investigated plants of the *Cucurbitaceae* family, zucchini showed more pronounced signs of toxicity than cucumber after sewage sludge application. The defensive reactions of zucchini plants are mainly associated with increases in peroxidase, APx, POx and GST activity. As the results of the lipid peroxide assay indicate a low level of oxidative damage in the tested plant tissues, the antioxidant system appears to function efficiently. However, further studies are needed to integrate knowledge about the activity and efficiency of the antioxidative system, and the detoxification reactions associated with the uptake of toxic compounds such as heavy metals and organic substances from sewage sludge.

## Supporting Information

S1 Supporting Information (XLS)

## References

[1] Cunningham SD, Anderson TA, Schwab AP, Hsu FC. Phytoremediation of soils contaminated with organic pollutants. Adv Agron. 1996; 56: 55–114.

[2] Mattina MJI, Iannucci-Berger W, Dykas L, Pardus J. Impact of long-term weathering, mobility, and land use on chlordane residues in soil. Env Sci Technol. 1999; 33 (14): 2425–2431.

[3] Nagajyoti PC, Lee KD, Sreekanth TVM. Heavy metals, occurrence and toxicity for plants: a review. Env Chem Lett. 2010; 8(3): 199–216

[4] Gworek B, Klimczak K, Kijeńska M. The relation between polyaromatic hydrocarbon concentration in sewage sludge and its uptake by plants: *Phragmites communis*, *Polygonum persicaria* and *Bidens tripartita*. PloS one. 2014; 9 (10): e109548 10.1371/journal.pone.0109548 25310699 PMC4195666

[5] Wyrwicka A, Steffani S, Urbaniak M. The effect of PCB-contaminated sewage sludge and sediment on metabolism of cucumber plants (*Cucumis sativus* L.). Ecohydrol Hydrobiol. 2014; 14 (1) 75–82.

[6] Liao Ch, Liang X, Lu G, Thai T, Xu W, Dang Zhi. Effect of surfactant amendment to PAHs-contaminated soil for phytoremediation by maize (*Zea mays* L.). Ecotox Environ Safe. 2015; 112: 1–6. 10.1016/j.ecoenv.2014.10.025 25463846

[7] Macek T, Mackova M, Kas J. Exploitation of plants for the removal of organics in environmental remediation. Biotechnol Adv. 2000; 18: 23–34. 14538117 10.1016/s0734-9750(99)00034-8

[8] Gent MPN, White JC, Parrish ZD, Isleyen M, Eitzer BD, Mattina MJI. Uptake and translocation of *p*,*p'*-dichlorodiphenyldichloroethylene supplied in hydroponics solution to *Cucurbita*. Environ Toxicol Chem. 2007; 26(12): 2467–2475. 18020671 10.1897/06-257.1

[9] Gerhardt KE, Huang X-d, Glick BR, Greenberg BM. Phytoremediation and rhizoremediation of organic soil contaminants: Potential and challenges. Plant Sci. 2009; 176: 20–30.

[10] Ivanov VB, Bystrova EI, Seregin IV. Comparative impacts of heavy metals on root growth as related to their specificity and selectivity. Russ J Plant Physiol. 2003; 50 (3): 398–406.

[11] Reddy AM, Kumar SG, Jyothsnakumari G, Thimmanaik S, Sudhakar Ch. Lead induced changes in antioxidant metabolism of horsegram (*Macrotyloma uniflorum* (Lam.) Verdc.) and bengalgram (*Cicer arietinum* L.). Chemosphere. 2005; 60(1): 97–104. 15910908 10.1016/j.chemosphere.2004.11.092

[12] Lyubenova L, Nehnevajova E, Herzig R, Schröder P. Response of antioxidant enzymes in *Nicotiana tabacum* clones during phytoextraction of heavy metals. Environ Sci Pollut R. 2009; 16 (5): 573–581. 10.1007/s11356-009-0175-8 19440744

[13] Briggs GG, Bromilow RH, Evans AA. Relationships between lipophilicity and root uptake and translocation of non-ionised chemicals by barley. Pestic Sci. 1982; 13 (5): 495–504.

[14] Hatzinger PB, Alexander M. Effect of aging of chemicals in soil on their biodegradability and extractability. Environ Sci Technol. 1995; 29 (2): 537–545. 10.1021/es00002a033 22201403

[15] Hülster A, Müller JF, Marschner H. Soil-plant transfer of polychlorinated dibenzo-*p*-dioxins and dibenzofurans to vegetables of the cucumber family (*Cucurbitaceae*). Environ Sci Technol. 1994; 28 (6): 1110–1115. 10.1021/es00055a021 22176237

[16] Mattina MI, Eitzer BD, Iannucci-Berger W, Lee WY, White JC. Plant uptake and translocation of highly weathered, soil-bound technical chlordane residues: Data from field and rhizotron studies. Environ Toxicol Chem. 2004; 23 (11): 2756–2762. 15559292 10.1897/03-570

[17] Parrish ZD, White JC, Isleyen M, Gent MPN, Iannucci-Berger W, Eitzer BD, et al Accumulation of weathered polycyclic aromatic hydrocarbons (PAHs) by plant and earthworm species. Chemosphere. 2006; 64 (4): 609–618. 16337258 10.1016/j.chemosphere.2005.11.003

[18] Engwall M, Hjelm K. Uptake of dioxin-like compounds from sewage sludge into various plant species–assessment of levels using a sensitive bioassay. Chemosphere. 2000; 40 (9–11): 1189–1195. 10739061 10.1016/s0045-6535(99)00368-9

[19] Mattina MJI, Iannucci-Berger W, Dykas L. Chlordane uptake and its translocation in food crops. J Agr Food Chem. 2000; 48 (5): 1909–1915. 10820114 10.1021/jf990566a

[20] White JC. Differential bioavailability of field-weathered *p*,*p′*-DDE to plants of the *Cucurbita* and *Cucumis* genera. Chemosphere. 2002; 49 (2): 143–152. 12375861 10.1016/s0045-6535(02)00277-1

[21] White JC, Wang X, Gent MPN, Iannucci-Berger W, Eitzer BD, Schultes NP, et al Subspecies-level variation in the phytoextraction of weathered *p*,*p'*-DDE by *Cucurbita pepo*. Environ Sci Technol. 2003; 37 (19): 4368–4373. 14572087 10.1021/es034357p

[22] White JC, Parrish ZD, Isleyen M, Gent MPN, Iannucci-Berger W, Eitzer BD, et al Uptake of weathered *p*,*p′*-DDE by plant species effective at accumulating soil elements. Microchem J. 2005; 81 (1): 148–155.

[23] Inui H, Wakai T, Gion K, Kim YS, Eun H. Differential uptake for dioxin-like compounds by zucchini subspecies. Chemosphere. 2008; 73 (10): 1602–1607. 10.1016/j.chemosphere.2008.08.013 18835616

[24] Zhang H, Chen J, Ni Y, Zhang Q, Zhao L. Uptake by roots and translocation to shoots of polychlorinated dibenzo-*p*-dioxins and dibenzofurans in typical crop plants. Chemosphere. 2009; 76 (6): 740–746. 10.1016/j.chemosphere.2009.05.030 19541345

[25] Low JE, Whitfield Åslund ML, Rutter A, Zeeb BA. Effect of plant age on PCB accumulation by *Cucurbita pepo* ssp. *pepo*. J Environ Qual. 2010; 39 (1): 245–50. 10.2134/jeq2009.0169 20048312

[26] White JC. Inheritance of *p*,*p*’-DDE phytoextraction ability in hybridized *Cucurbita pepo* cultivars. Environ Sci Technol. 2010; 44 (13): 5165–5169. 10.1021/es100706t 20507162

[27] Whitfield Åslund ML, Lunney AI, Rutter A, Zeeb BA. Effects of amendments on the uptake and distribution of DDT in *Cucurbita pepo* ssp. *pepo* plants. Environ Pollut. 2010; 158 (2): 508–513. 10.1016/j.envpol.2009.08.030 19762136

[28] Greenwood SJ, Rutter A, Zeeb BA. The absorption and translocation of polychlorinated biphenyl congeners by *Cucurbita pepo* ssp. *pepo*. Environ Sci Technol. 2011; 45 (15): 6511–6516. 10.1021/es200598u 21696136

[29] Low JE, Åslund MLW, Rutter A, Zeeb BA. The effects of pruning and nodal adventitious roots on polychlorinated biphenyl uptake by *Cucurbita pepo* grown in field conditions. Environ Pollut. 2011; 159 (3): 769–775. 10.1016/j.envpol.2010.11.015 21168941

[30] Matsuo S, Yamazaki K, Gion K, Eun H, Inui H. Structure-selective accumulation of polychlorinated biphenyls in *Cucurbita pepo*. J Pestic Sci. 2011; 36: 363–369.

[31] Antolín MC, Pascual I, García C, Polo A, Sánchez-Díaz M. Growth, yield and solute content of barley in soils treated with sewage sludge under semiarid Mediterranean conditions. Field Crop Res. 2005; 94 (2–3): 224–237.

[32] Tejada M, Gómez I, Fernández-Boy E, Díaz M-J. Effects of sewage sludge and *Acacia dealbata* composts on soil biochemical and chemical properties. Commun Soil Sci Plan. 2014; 45 (5): 570–580.

[33] Lakhdar A, ben Achiba W, Montemurro F, Jedidi N, Abdelly Ch. Effect of municipal solid waste compost and farmyard manure application on heavy-metal uptake in wheat. Commun Soil Sci Plan. 2009; 40 (21–22): 3524–3538.

[34] Waqas M, Khan S, Qing H, Reid BJ, Chao C. The effects of sewage sludge and sewage sludge biochar on PAHs and potentially toxic element bioaccumulation in *Cucumis sativa* L. Chemosphere. 2014; 105: 53–61. 10.1016/j.chemosphere.2013.11.064 24360844

[35] Gonçalves JF, Becker AG, Pereira LB, Rocha JBT, Cargnelutti D, Tabaldi LA, et al Response of *Cucumis sativus* L. seedlings to Pb exposure. Braz J Plant Physiol. 2009; 21 (3): 175–186.

[36] Hernández JA, Campillo A, Jiménez A, Alarcón JJ, Sevilla F. Response of antioxidant systems and leaf water relations to NaCl stress in pea plants. New Phytol. 1999; 141: 241–251. 10.1046/j.1469-8137.1999.00341.x 33862919

[37] Dai Q, Yan B, Huang S, Liu X, Peng S, Miranda MLL, et al Response of oxidative stress defense systems in rice (*Oryza sativa*) leaves with supplemental UV-B radiation. Physiol Plant. 1997; 101: 301–308.

[38] Velikova V, Yordanow I, Edreva A. Oxidative stress and some antioxidant systems in acid rain-treated bean plants. Protective role of exogenous polyamines. Plant Sci. 2000; 151: 59–66.

[39] Wyrwicka A, Skłodowska M. Influence of repeated acid rain treatment on antioxidative enzyme activities and on lipid peroxidation in cucumber leaves. Environ Exp Bot. 2006; 56 (2): 198–204.

[40] Wyrwicka A, Skłodowska M. Intercompartmental differences between cytosol and mitochondria in their respective antioxidative responses and lipid peroxidation levels in acid rain stress. Acta Physiol Plant. 2014; 36 (4): 837–848.

[41] Mittler R. Oxidative stress, antioxidants and stress tolerance. Trends Plant Sci. 2002; 7: 405–410. 12234732 10.1016/s1360-1385(02)02312-9

[42] Møller IM. Plant mitochondria and oxidative stress: electron transport, NADPH turnover, and metabolism of reactive oxygen species. Annu Rev Plant Physiol Plant Mol Biol. 2001; 52: 561–591. 11337409 10.1146/annurev.arplant.52.1.561

[43] Dhindsa RS, Plumb-Dhindsa P, Thorpe TA. Leaf senescence: correlated with increased levels of membrane permeability and lipid peroxidation, and decreased levels of superoxide dismutase and catalase. J Exp Bot. 1981; 32: 93–101.

[44] Willekens H, Inzé D, Van Montagu M, Van Camp W. Catalases in plants. Mol Breed. 1995; 1: 207–228.

[45] Jiménez A, Hernández JA, del Río LA, Sevilla F. Evidence for the presence of the ascorbate–glutathione cycle in mitochondria and peroxisomes of pea leaves. Plant Physiol. 1997; 114: 275–284. 12223704 10.1104/pp.114.1.275 PMC158303

[46] Tayefi-Nasrabadi H, Dehghan G, Daeihassani B, Movafegi A, Samadi A. Some biochemical properties of guaiacol peroxidases as modified by salt stress in leaves of salt-tolerant and salt-sensitive safflower (*Carthamus tinctorius L*.*cv*.) cultivars. Afr J Biotech. 2011; 10: 751–763.

[47] Noctor G, Foyer CH. Ascorbate and glutathione: keeping active oxygen under control. Annu Rev Plant Phys. 1998; 49: 249–279. 10.1146/annurev.arplant.49.1.249 15012235

[48] Tausz M, Grill D. The role of glutathione in stress adaptation of plants. Phyton. 2000; 40: 111–118.

[49] Karolewski P, Giertych MJ. Changes in the level of phenols during needle development in Scots pine populations in a control and polluted environment. Eur J For Pathol. 1995; 25: 297–306.

[50] Schafer RQ, Wang HP, Kelley EE, Cueno KL, Martin SM, Buettner GR. Comparing β-carotene, vitamin E and nitric oxide as membrane antioxidants. Biol Chem. 2002; 383: 671–681. 12033456 10.1515/BC.2002.069

[51] Edwards R, Dixon DP. Metabolism of natural and xenobiotic substances by the plant glutathione S-transferase superfamily In: Sandermann H, editor. Ecological Studies vol. 170, Molecular Ecotoxicology of Plants. Berlin Heidelberg: Springer-Verlag; 2004 pp. 17–50.

[52] Marrs KA. The functions and regulation of glutathione S-transferases in plants. Annu Rev Plant Phys. 1996; 47: 127–158. 10.1146/annurev.arplant.47.1.127 15012285

[53] Nakano Y, Asada K. Hydrogen peroxide is scavenged by ascorbate-specific peroxidase in spinach chloroplasts. Plant Cell Physiol. 1981; 22: 867–880.

[54] Habig WH, Pabst MJ, Jakoby WB. Glutathione S-transferases. The first enzymatic step in mercaptane acid formation. J Biol Chem. 1974; 246: 7130–7139. 4436300

[55] Maehly AC, Chance B. The assay of catalases and peroxidases In: Glick D, editor. Methods of Biochemical Analysis Vol. 1. New York: Interscience Publishers Inc; 1954 pp. 357–425. 10.1002/9780470110171.ch14 13193536

[56] Yagi K. Assay for serum lipid peroxide level its clinical significance In: Yagi K, editor. Lipid Peroxides in Biology and Medicine. London, New York: Acad Press Inc; 1982 pp. 223–241.

[57] Bradford MM. A rapid and sensitive method for the quantification of microgram quantities of protein utilizing the principle of protein-dye binding. Anal Biochem. 1976; 72: 248–254. 942051 10.1016/0003-2697(76)90527-3

[58] Taylor SL, Tappel AL. Sensitive fluorometric methods for tissue tocopherol analysis. Lipids 1976; 11: 530–538. 948248 10.1007/BF02532898

[59] Clostre F, Letourmy P, Turpin B, Carles C, Lesueur-Jannoyer M. Soil type and growing conditions influence uptake and translocation of organochlorine (chlordecone) by Cucurbitaceae species. Water Air Soil Pollut. 2014; 225 (10): 2153.

[60] Mattina MI, Lannucci-Berger W, Musante C, White JC. Concurrent plant uptake of heavy metals and persistent organic pollutants from soil. Environ Pollut. 2003; 124 (3): 375–378. 12758018 10.1016/s0269-7491(03)00060-5

[61] Mitton FM, Ribas Ferreira JL, Gonzalez M, Miglioranza KSB, Monserrat JM. Antioxidant responses in soybean and alfalfa plants grown in DDTs contaminated soils: Useful variables for selecting plants for soil phytoremediation? Pestic Biochem Phys. 2015; 10.1016/j.pestbp.2015.12.005 27155479

[62] Rutherford AW, Krieger-Liszkay A. Herbicide-induced oxidative stress in photosystem II. Trends Biochem Sci. 2001; 26 (11): 648–653. 11701322 10.1016/s0968-0004(01)01953-3

[63] Antolín MC, Muro I, Sánchez-Díaz M. Sewage sludge application can induce changes in antioxidant status of nodulated alfalfa plants. Ecotox Environ Safe. 2010; 73: 436–442. 10.1016/j.ecoenv.2009.08.022 19959231

[64] Martí MC, Camejo D, Fernández-García N, Rellán-Alvarez R, Marques S, Sevilla F, et al Effect of oil refinery sludges on the growth and antioxidant system of alfalfa plants. J Hazar Mater. 2009; 171 (1–3): 879–885. 10.1016/j.jhazmat.2009.06.083 19596515

[65] Antolín MC, Muro I, Sánchez-Díaz M. Application of sewage sludge improves growth, photosynthesis and antioxidant activities of nodulated alfalfa plants under drought conditions. Environ Exp Bot. 2010; 68: 75–82.

[66] Wan L, Peijun L, Qixing Z, Tieheng S, Peidong T, Huaxia X. Short-term toxic effects of chlorobenzenes on broadbeen (*Vicia faba*) seedlings. Sci China Ser C. 2005; 48 (1): 33–39. 10.1007/BF02889799 16089327

[67] Pašková V, Hilscherová K, Feldmanová M, Bláha L. Toxic effects and oxidative stress in higher plants exposed to polycyclic aromatic hydrocarbons and their *N*-heterocyclic derivatives. Environ Tox Chem. 2006; 25 (12): 3238–3245. 10.1897/06-162r.1 17220094

[68] Liu H, Weisman D, Ye YB, Cui B, Huang YH, Colón-Carmona A, et al An oxidative stress response to polycyclic aromatic hydrocarbon exposure is rapid and complex in *Arabidopsis thaliana*. Plant Sci. 2009; 176 (3): 375–382.

[69] Hanano A, Almousally I, Shaban M. Phytotoxicity effects and biological responses of *Arabidopsis thaliana* to 2,3,7,8-tetrachlorinated dibenzo-*p*-dioxin exposure. Chemosphere. 2014; 104: 76–84. 10.1016/j.chemosphere.2013.10.060 24275148

[70] Xu J, Su ZH, Chen C, Han HJ, Zhu B, Fu XY, et al Stress responses to phenol in *Arabidopsis* and transcriptional changes revealed by microarray analysis. Planta. 2012; 235 (2): 399–410. 10.1007/s00425-011-1498-5 21927950

[71] Bartosz G. Oxidative stress in plants. Acta Physiol Plant. 1997; 19: 47–64.

[72] Scott D. Catalase In: Reed G, editor. Enzymes in food processing. Second edition New York, San Francisco, London: Academic Press; 1975.

[73] Switala J, Loewen PC. Diversity of properties among catalases. Arch Biochem Biophys. 2002; 401: 145–154. 12054464 10.1016/S0003-9861(02)00049-8

[74] Levine A, Tenhaken R, Dixon R, Lamb C. H_2_O_2_ from the oxidative burst orchestrates the plant hypersensitive disease resistance response. Cell. 1994; 79: 583–593. 7954825 10.1016/0092-8674(94)90544-4

[75] Ludwikow A, Gallois P, Sadowski J. Ozone-induced oxidative stress response in *Arabidopsis*: transcription profiling by microarray approach. Cell Mol Biol Lett. 2004; 9 (4B): 829–42. 15647800

[76] Schützendübel A, Nikolova P, Rudolf C, Polle A. Cadmium and H_2_O_2_-induced oxidative stress in *Populus* × *canescens* roots. Plant Physiol Biochem. 2002; 40 (6–8): 577–584.

[77] Kang H-M, Saltveit ME. Activity of enzymatic antioxidant defence systems in chilled and heat shocked cucumber seedling radicles. Physiol Plant. 2001; 113: 548–556.

[78] Zhu Z, Wei G, Li J, Qian Q, Yu J. Silicon alleviates salt stress and increases antioxidant enzymes activity in leaves of salt-stressed cucumber (*Cucumis sativus* L.). Plant Sci. 2004; 167: 527–533.

[79] Boo YC, Jung J. Water deficit–induced oxidative stress and antioxidative defenses in rice plants. J Plant Physiol. 1999; 155: 255–261.

[80] Tanaka K, Otsubo T, Kondo N. Participation of hydrogen peroxide in the inactivation of Calvin-cycle SH enzymes in SO_2_-fumigated spinach leaves. Plant Cell Physiol. 1982; 23: 1009–1018.

[81] Feierabend J, Kemmerich P. Mode of interference of chlorosis-inducing herbicides with peroxisomal enzyme activities. Physiol Plant. 1983; 57: 346–351.

[82] Gabara B, Skłodowska M, Wyrwicka A, Glińska S, Gapińska M. Changes in the ultrastructure of chloroplasts and mitochondria and antioxidant enzyme activity in *Lycopersicon esculentum* Mill. leaves sprayed with acid rain. Plant Sci. 2003; 164: 507–516.

[83] Roy S, Lindström-Seppä P, Huuskonen S, Hänninen O. Responses of biotransformation and antioxidant enzymes in *Lemna minor* and *Oncorhynchus mykiss* exposed simultaneously to hexachlorobenzene. Chemosphere. 1995; 30 (8): 1489–1498.

[84] Pascal S, Scalla R. Purification and characterization of a safener-induced glutathione S-transferase from wheat (*Triticum aestivum*). Physiol Plant. 1999; 106 (1): 17–27.

[85] Tang J, Hoagland KD, Siegfried BD. Uptake and bioconcentration of atrazine by selected freshwater alga. Environ Toxicol Chem. 1998; 17 (6): 1085–1090.

[86] Hamoutène D, Roméo M, Gnassia M, Lafaurie M. Cadmium effects on oxidative metabolism in a marine seagrass: *Posidonia oceanica*. Bull Environ Contam Toxicol. 1996; 56: 327–334.

[87] Ranvier S, Gnassia-Bareli M, Pergent G, Capiomont A, Roméo M. The effect of mercury on glutathione S-transferase in the marine phanerogam Posidonia oceanica. Bot Mar. 2000; 43: 161–168.

[88] Ferrat L, Pergent-Martini C, Roméo M. Assessment of the use of biomarkers in aquatic plants for the evaluation of environmental quality: application to seagrasses. Aquat Toxicol. 2003; 65 (2): 187–204. 12946618 10.1016/s0166-445x(03)00133-4

[89] Menone ML, Pflugmacher S. Effects of 3-chlorobiphenyl on photosynthetic oxygen production, glutathione content and detoxification enzymes in the aquatic macrophyte *Ceratophyllum demersum*. Chemosphere. 2005; 60 (1): 79–84. 15910905 10.1016/j.chemosphere.2004.11.094

